# Examining the Association Between Parental Socioeconomic Status and Preterm Birth Using Multidomain Social Determinants Scale in a Tertiary Care Center in Saudi Arabia

**DOI:** 10.7759/cureus.10506

**Published:** 2020-09-17

**Authors:** Mohammed Y Al-Hindi, Hazem Aljuhani, Anas R Alnajjar, Sarah Alessa, Mansour Alqurashi, Yaser A Faden

**Affiliations:** 1 Pediatrics, King Abdulaziz Medical City – Western Region, Ministry of National Guard, Jeddah, SAU; 2 College of Medicine, King Saud Bin Abdulaziz University for Health Sciences, Jeddah, SAU; 3 Research and Development, King Abdullah International Medical Research Center – Western Region, Jeddah, SAU; 4 College of Medicine, King Abdulaziz University Hospital, Jeddah, SAU; 5 Obstetrics and Gynecology, King Abdulaziz Medical City – Western Region, Ministry of National Guard, Jeddah, SAU

**Keywords:** preterm delivery, socioeconomic status, polysocial risk score, preterm birth, multidomain scale, birth weight, socioeconomic factors

## Abstract

Objectives

Socioeconomic status (SES) plays a conflicting role in preterm birth (PB). This study evaluated the association between SES and PB using, for the first time, a multidomain scale, validated for Saudi Arabia, with a scoring system and examined the effect of each SES domain on PB. The secondary outcome was to determine the effects of SES on birth weight (BW) and the subcategories of PB and BW.

Methods

This cross-sectional study was conducted between May 2017 and August 2017 at a National Guard tertiary center in Jeddah, Saudi Arabia. A total of 477 parents were interviewed using the Elzahrany R. SES scale.

Results

The rate of PB was 11.5%, with no significant differences among the high, middle, and low SES classes (13%, 11%, and 12.5%, respectively). There were no patients in the very low SES in this specific population. None of the maternal or neonatal characteristics were significantly different among SES classes except maternal age (p value = 0.03), and antenatal care recorded visits “booking” status (p value = 0.012). Stratified analysis for PB subcategories showed the lower SES classes had higher moderate (3.8%) and extreme (1.6%) PB. For BW subcategories, large for gestational age (LGA) infants were higher in the high SES class (13%). However, the lower SES classes had higher rates of lower BW. The association between SES and PB remained not significant after adjusting for the maternal age and antenatal booking status.

Conclusion

There was no association between SES and PB at a tertiary center providing universal care to the National Guard using multidomain socioeconomic determinants with a scoring system. However, lower SES was associated with lower BW. The use of the “polysocial risk score” based on locally validated surveys should be considered in any health research that examines the effects of socioeconomic determinants.

## Introduction

Preterm birth (PB), i.e., birth before 37 weeks of gestation, is a significant burden worldwide as it is the second direct cause of neonatal mortality. In a year, 15 million neonates are born preterm, and the incidence is higher in lower income countries than in high-income ones [1]. Approximately half of the PBs are idiopathic [2].

Recently, several studies revealed the significant role of socioeconomic status (SES) on different aspects of health, including physical and psychological, among all age groups in society [3]. SES is a reflective measurement of an individual’s positions in a community based on different factors such as education, income, and occupation. According to these factors, individuals are classified into social hierarchy [4]. Previous studies were conducted to investigate the effect of SES on prematurity. Some of these studies showed an association, while others did not [5-7]. These contrasting results may be explained by the differences in predictors and measurements used in each study.

Researchers have built several scales to include various factors that are determined by differences in culture and geographical location. In the United States and India, the most commonly used scales are Hollingshead and Kuppuswamy scales, respectively [8,9]. Moreover, the Fahmy and El-Sherbini and El-Shakhs (The scale manual. El-Shakhs AE. A scale of socioeconomic status of the family. Cairo, Anglo-Egyptian Stationery, 1995) scales are the only scales in the Middle East [10]. The former is generally used in health research, while the latter is in educational fields [11]. The updated and validated Fahmy and El-Sherbini scale categorizes families into five classes based on seven domains [11]. Elzahrany, a sociologist, formulated and validated a multidomain, socioeconomic determinant with a scoring system in Saudi Arabia, which consists of seven domains adopted from the Fahmy and El-Sherbini scale (Unpublished research. Elzahrany RA: Socio-economic Levels and Standards of Living in Makkah “Mecca”, Saudi Arabia. Geography Department, College of Social Sciences, Umm Al-Qura University, 2017).

To the best of our knowledge, no published study in Saudi Arabia has measured SES based on a scale and scoring system. Moreover, the association between SES and prematurity in the region has not yet been elucidated. Thus, this study aims to evaluate the association between SES and PB using a validated multidomain scale (Elzahrany R. scale) with a scoring system validated for Saudi Arabia. The secondary objectives are to examine the effect of each SES domain on PB and determine the low-birth-weight (LBW) distribution among SES classes.

## Materials and methods

This cross-sectional study was conducted in pre- and postnatal care units and neonatal intensive care unit (NICU) of King Abdulaziz Medical City (KAMC), Ministry of National Guard, Western Region, Jeddah, Saudi Arabia. The survey period was between May 2017 and August 2017. The study followed the principles of the Helsinki Declaration, and Institutional Review Board (IRB) approval was obtained from the IRB of King Abdulla International Medical Research Center. The study participants included all parents of newborns in these care units. On the other hand, parents who refused to participate, had multiple pregnancies (more than two kids), or had newborns diagnosed with severe/chromosomal congenital anomalies were excluded from the study.

A sample size of 477 was calculated based on a population data that calculated the prematurity rate 11.7% in unit audit in 2015, an estimated incidence 6.5% of prematurity rate in the lower SES, a level of significance of 5%, and a power of 80% to detect a statistically significant difference.

A non-probability consecutive sampling technique was used in which all included were identified from the admission logbooks in the expected wards. The participants were categorized into “case,” those who had PB, and control groups, those with term (≥37 weeks of gestation) birth. After obtaining consent, the primary household was interviewed directly or by telephone for the socioeconomic determinant data. Subsequently, we collected the parental and neonatal basic demographics in addition to the designation of newborns from hospital health electronic records. PB was subcategorized into late (LPB, birth between 34 and 36 weeks), moderate (MPB, birth between 28 and 33 weeks), and extreme (EPB, birth between 23 and 27 weeks). Birth weight (BW) was subcategorized into large (LBW, birth weight > 4,000), normal (birth weight of 2,500-4,000 g), low (LBW, birth weight of 2,500-1,500 g) very low (VLBW, birth weight of 1,000-1,499 g), and extreme (ELBW, birth weight < 1,000 g).

The questionnaire developed by Elzahrany was used to gather the socioeconomic details. It aimed to measure SES via a validated scoring system that fits Saudi Arabia. Elzahrany’s team was composed of a sociologist and epidemiologist and medical professionals experienced in the field and social studies. This scale also consists of four main domains, which were adopted from the Fahmy and El-Sherbini scale [11]: (1) residential characteristics; (2) demographics that included four sub-domains: the number of family members, education, occupation, and income of both parents; (3) tourism and entertainment; and (4) health status of family members. This questionnaire was collected via two separate field surveys conducted in 2016 and 2017 in which the participants were selected via a random cluster sample and the results were validated through kappa statistics, which showed strong agreement. Factor analysis was used to calculate the scores. We did not need to validate language as both surveys, Elzahrani R. and Fahmy, were in Arabic. SES was then categorized into four classes (high, middle, low, and very low) based on the 25th segment increments of the participant’s scores (see attached Appendix).

Data were collected via a Google form that generated an Excel sheet of raw data and were screened for any missing data and outliers. Quantitative data were described by central tendency (mean and standard deviation [SD], median and interquartile range [IQR]) and graphs (histogram and box plots), whereas frequency tables and graphs (bar charts) were for qualitative data. The student’s t-test or the median test was performed to compare quantitative data accordingly. On the other hand, chi-square/Fisher’s exact test was used for the comparisons of qualitative data. A p value of <0.05 was considered significant. Data were analyzed using the IBM® SPSS® Version 24 (Armonk, NY, IBM Corp.). Stratified and logistic regression analyses were planned to assess confounding factors.

## Results

A total of 987 participants were recruited in the study. Among these, only 485 (49%) responded, of which 33 (6.8%) refused to be included and 10 were excluded as per the exclusion criteria. Therefore, we interviewed 442 parents. The mean SES score was 55.4 (±SD 10) and a median of 54 (50% IQR 49-61). There were 120 participants (27.1%) who were scored between 25 and 49 (low SES status level) with a median of 46 (IQR 43-48). On the other hand, 299 (76.5%) subjects were scored between 50 and 74 (middle SES level) with a median of 56 (IQR 53-62). Only 23 (5.2%) participants were scored 75 or more (high SES level) with a median of 78 (50% IQR 76-79). Finally, no participants were scored <25 (very low SES class); therefore, this class was not analyzed.

In maternal demographics, the mean age of the mothers was 31.4 ± 5.4 years in the high class compared with 28.2 ± 5.3 and 28.4 ± 6.4 in the middle and low classes, respectively (p value = 0.033). Moreover, the number of booked mothers (pregnant with a recorded regular antenatal follow-up) was higher in the middle class with a percentage of 84.9% compared with 77.3% and 72.5% in the high and low classes, respectively (p value = 0.012). The remaining maternal demographics showed no statistically significant differences were noted among the classes. The pregnancy-related disease was more common in the high class but was not statistically significant. In neonatal demographics, BW, gestational age, cord pH, and Apgar score were not statistically significantly different. There was a trend toward less small for gestation and admission to NICU in the high class, although it was not statistically significant (Table 1).

**Table 1:** Maternal and neonatal clinical characteristics among SES classes M, mean; SD, standard deviation

Characteristics	Low class (n = 120)	Middle class (n = 299)	High class (n = 23)	p values	
Maternal					
Age (M ± SD)	28.4 ± 6.4	28.2 ± 5.3	31.4 ± 5.4	0.033	
Height in cm (M ± SD)	156.1 ± 5.7	156.9 ± 6.0	158.8 ± 5.5	0.110	
Weight in kg (M ± SD)	72.1 ± 16.8	72.7 ± 14.3	75.1 ± 13.9	0.675	
Gestational diabetes (%)	16.9%	12.4%	27.3%	0.120	
Gestational hypertension (%)	4.2%	5.4%	9.1%	0.463	
Booking status (% booked)	72.5%	84.9%	77.3%	0.012	
Neonatal					
Gender (% male)	42.5%	52.2%	56.5%		0.161
Birth weight in g (M ± SD)	2879.5 ± 694.9	3061.2 ± 1471.9	3277.4 ± 634.9		0.259
Gestational age (M ± SD)	38.0 ± 3.2	38.6 ± 2.2	38.7 ± 1.6		0.058
Small for gestational age (%)	12.5%	15.1%	4.3%		0.318
*Cord pH (M ± SD)	7.2 ± 0.08	7.2 ± 0.08	7.3 ± 0.07		0.290
Apgar score (median, range)	9.0, 7.0	9.0, 10.0	9.0, 4.0		NA
Admission to NICU (%)	11.9%	7.4%	4.3%		0.316

Among the 442 mothers, 391 (88.5%) had term delivery and 51 (11.5%) had preterm delivery. There was no difference in SES scores between those who had term or PB [median 54 (50% IQR 49-61) vs. 55 (50% IQR 48-62)]. The rate of PB was 11.5% among all participants, and no significant difference was noted among the SES classes, with PB rates of 12.5%, 11%, and 13% for low, middle, and high SES classes, respectively (p value = 0.9).

In the stratified analysis for further subcategories of PBs, a trend toward an increase in late PB was noted (13%) in the high SES class compared with low and middle classes. In contrast, lower SES classes had higher moderate and extreme PB (4.2% and 3.3%), respectively, however, not statistically significant (p value = 0.9). The same analysis for BW categories showed that infants with LBW were higher in the high SES class. However, the lower SES classes had higher rates of VLBW and ELBW (p value = 0.016) (Table 2).

**Table 2:** Gestational age and birth weight stratified analysis *p value = 0.9 **p value = 0.016

Categories	SES Class			
	Low	Middle	High	Total
Gestational age, weeks*				
Term	105 (87.5%)	266 (89%)	20 (87%)	391 (88.5%)
Late preterm, 34–36	6 (5%)	25 (8.4%)	3 (13%)	34 (7.7%)
Moderate preterm, 28–33	5 (4.2%)	7 (2.3%)	0 (0%)	12 (2.7%)
Extreme preterm, <28	4 (3.3%)	1 (0.3%)	0 (0%)	5 (1.1%)
Total	120 (27.15%)	299 (67.65%)	23 (5.20%)	442 (100%)
Birth weight, grams **				
Normal BW	94 (78.3%)	251 (84.2%)	17 (73.9%)	362 (82.1%)
Large BW, >4000	3 (2.5%)	4 (1.3%)	3 (13%)	10 (2.3)
Low BW, 1500–2499	15 (12.5%)	39 (13.1%)	3 (13%)	57 (12.9%)
Very low BW, 1000–1499	3 (2.5%)	1 (0.3%)	0 (0%)	4 (0.9%)
Extremely low BW, <1000	5 (4.2%)	3 (1%)	0 (0%)	8 (1.8%)
Total	120 (27.21%)	298 (67.57%)	23 (5.22%)	441 (100%)

We further examined the significant risk factors detected via univariate analysis, namely maternal age and antenatal care booking status. In logistic regression analysis, the association between PB and SES classes was not significant even after adjusting for the included risk factors.

## Discussion

Our study did not find an association between SES and PB. We obtained a prematurity rate of 11.5% among all deliveries in KAMC, which is similar to the international figures (10.6%) but higher than the national numbers (3.9%). This could be explained by the fact that KAMC is considered a tertiary center and therefore accepts higher risk pregnant women.

In stratified analysis, SES was also not associated with PB subclasses (late, intermediate, and extreme). However, BW in the stratified analysis showed that higher SES was associated with LGA. In addition, LBW and VLBW were associated with lower SES classes.

Our study concurs with the Canadian study by Campbell et al. [12]. They examined the effect of the following multiple socioeconomic variables: maternal education, income, immigration prevalence, and minority prevalence on multiple birth adverse outcomes including PB. Socioeconomic data were extracted from the 2011 Canadian Census. None of these socioeconomic variables were significantly associated with PB except for immigration and visible minority prevalence. However, our results are conflicting with those in the study by Parker et al. that used data from the National Maternal and Infant Health Survey by the National Center for Health Statistics [6]. They investigated the effect of multiple socioeconomic domains on PB, LBW, and small for gestational age. Regarding PB, lower maternal and paternal education levels among the black ethnic groups, in addition to certain maternal occupations in both black-and-white ethnic groups, were significantly associated with higher odds of PB. Furthermore, another Italian population-based study by Cantarutti et al. examined the effect of maternal education on several neonatal outcomes [5]. The results showed that a higher maternal education level was significantly associated with reduced odds of PB.

Regarding BW, in the study by Campbell et al., a weak association was noted between maternal education and LBW [12]. However, none of the other socioeconomic variables were associated with LBW. Further analysis was performed using birth percentiles (<5th and <10th) and showed that a higher population density was associated with increased odds of birth of <10th percentile. On the other hand, Parker et al. found that lower education levels among black mothers and both black-and-white fathers, in addition to certain maternal occupations in black mothers and low income in both ethnic groups, were significantly associated with higher odds of LBW [6]. This is also consistent with the results in the study by Cantarutti et al. [5]. In our study, we had similar findings as VLBW and ELBW incidences were higher in the low SES class after controlling other biological causes. The high SES class had a higher prevalence of LBW infants, which was not reported in comparative studies [5,6,12]. This can be explained by the higher prevalence of gestational diabetes in the high SES class.

All of the above population-based studies showing variable results could be explained by varying reasons. First, different populations with different racial and ethnic backgrounds could contribute to PB. The above examples are of high-income countries. Our study, although local, can highlight the effect of SES on PB on a specific population. This ignites the need for a large population-based national study. Second, the above studies independently examined the effects of different domains of SES. This approach has been recently questioned [13]. Figueroa et al. recommend using the “polysocial risk score” where socioeconomic domains are interlinked and should be taken as a whole rather than as independent segments [13]. To the best of our knowledge, our study is the first that studied the effect of SES on PB using a similar concept with a scoring system that aggregates socioeconomic domains and calculates the final scores.

This is the first national study that examined the effect of SES on PB using a validated multidomain scoring system that reflected our population. It included National Guard soldiers and their families. Those were usually “booked,” i.e., followed up regularly in the antenatal clinics. However, approximately 25% of our population is “not-booked” either due to ineligibility or being accepted as a referral high-risk case, both representing non-military families. Our population was all employed and had universal access to primary and tertiary health-care facilities. Thus, no participants fell under the very low class of SES. Our results are more consistent with those in the Canadian study as both countries have a universal education and health system.

Previous studies discussed the biological pathways leading to PB through neuroendocrine processes precipitated by stressful experiences. These stressful conditions included poor nutrition, prepregnancy health status, lack of medical care, social isolation, and workplace. Our sample did not reflect these stressful living conditions as well as the lack of very low socioeconomic class in the study [14,15].

The limitation of our study is that this is a single-center study reflecting the special population of National Guard soldiers and their families. Hence, our results are reliable but mainly for military/National Guard personnel. However, this could indirectly reflect the effect of a universal health-care system on a targeted population. A low sample size could underestimate the stratified analysis; moreover, the nature of the population did not include ethnic variation data as a factor. Recall bias could affect our results as any survey-based study.

## Conclusions

In conclusion, using a multidomain-validated scoring system to measure SES in Saudi Arabia in a specific population, we did not find an association between SES and PB even with further stratified analysis. However, the SES effect on BW, the stratified analysis showed that the higher SES class was associated with LGA, whereas LBW and VLBW were associated with the lower SES classes. Thus, we recommend conducting a large multicenter cluster random sampling or population-based study to have a better representation and include the very low SES class. Finally, the use of the “polysocial risk score” based on locally validated surveys should be considered in any health research that examines the effects of socioeconomic determinants.

## Appendices

**Table 3:** Elzahrany SES score

This scale includes four domains with a total score of 97. Socioeconomic level: to be classified into very low, low, middle, high levels depending on the quartile of the score calculated						
Residential characteristics:						
Type of residency (Score = 3):		Place of residence classification (Score = 1):			Number of the rooms (Score = 4):	
Villa	3	Slums	0		4 and more	4
Apartment	2	Residential plans	1		Less than 4	3
Traditional house	1					
Property ownership (Score = 4):		House services (Score = 4):			Car ownerships (Score = 2):	
Owned	4	Connected to electrical grid	1		Doesn’t have a car	0
Given from the employer	3	Connected to water pipe system	1		One car	1
Rent	2	Connected to internet network	1		Multiple cars	2
Others (e.g., charity)	1	Connected to sanitation	1			
Property at home (Score = 7:1 each for the presence of items given below)						
Telephone line, Cellphone, TV, Radio, Refrigerator, Washing machine, Computer						
Demographics:						
Number of family living in the house (Score = 3):		Educational level of the children > 6 years old (Score = 3):			Number of house workers (Score = 2):	
3 or less	3	All in schools, universities	3		None	0
4-6	2	Half and more in schools, universities	2		One	1
7-9	1	Less than half in schools, universities	1		Two or more	2
10 and more	0	No one attended the schools	0			
Number of family member who have income (Score = 3):			Income adequacy (Score = 3):			
1	1		Not sufficient for necessary needs		0	
2	2		Sufficient for necessary needs		1	
3 and more	3		Sufficient for necessary and emergency needs		2	
			Enough for saving and investment		3	
Family monthly income by SAR (Score = 16):		Educational level (Score = 26):			Occupational status (Score = 10):	
No income at all	0		Husband	Wife		Husband Wife
Less than 2500	2	Illiterate	0	0	Not working/housewife	0 0
2500-4999	4	Read and write	2	2	Unskilled manual worker	1 1
5000-7499	6	Elementary school	4	4	Skilled manual worker	2 2
7500-9999	8	Secondary school	6	6	Business	3 3
10000-12499	10	High school	6	6	Semi-professional like	
12500-14999	12	Bachelor	10	10	(general education)	4 4
15000-19999	14	Master/PhD	13	13	Professional like	
20000 and more	16				(physician, engineering…)	5 5
Tourism and entertainment:						
Spend vacation not less than one week inside Saudi Arabia in the past two years:		Spend vacation not less than one week in an Arabic country in the past two years:			Eating from restaurants in the past two weeks:	
				No		0	No	0		No	0
Yes	1	Yes	2		Yes	1
Family members health status:						
Family members who have chronic disease or disabilities			No 1	Yes 0		
Infant mortality before completing one year of age last year			No 1	Yes 0		
